# Preeclampsia and environmental epigenomics: the emerging role of air pollution, gut microbiome, and maternal exposures in disease programming

**DOI:** 10.1093/eep/dvag001

**Published:** 2026-01-10

**Authors:** Geethika Yelleti, Nihaal Maripini, Varashree Bolar Suryakanth

**Affiliations:** Department of Biochemistry, Kasturba Medical College, Manipal Academy of Higher Education, Manipal, Karnataka 576104, India; Department of Biochemistry, Kasturba Medical College, Manipal Academy of Higher Education, Manipal, Karnataka 576104, India; Department of Biochemistry, Kasturba Medical College, Manipal Academy of Higher Education, Manipal, Karnataka 576104, India

**Keywords:** preeclampsia, environmental epigenomics, air pollution, gut microbiome, microRNA, DNA methylation, precision medicine

## Abstract

Preeclampsia (PE) remains a major contributor of maternal and fetal morbidity and mortality worldwide, affecting 2%–8% of pregnancies. While genetic predisposition, placental dysfunction, and angiogenic imbalance remain central to PE pathophysiology, emerging observational evidence suggests potential associations between environmental factors, epigenetic modifications, and PE development. This review consolidates available research linking environmental exposures, particularly air pollution, maternal gut microbiome composition, and dietary habits, with changes in epigenetic markers during pregnancy that may influence PE susceptibility. We synthesize findings from epidemiological studies, mechanistic research, and biomarker studies across this research area. However, definitive causal evidence linking specific environmental exposures to PE through epigenetic mechanisms remains limited. The majority of existing studies employ observational designs or focus on biological mechanisms; well-designed prospective cohorts incorporating direct environmental measurements and randomized intervention trials are lacking. Circulating biomarkers, including microRNAs and DNA methylation patterns, show associations with both PE status and prior environmental exposure, providing biological support for the concept that environmental factors may influence PE development. The maternal gut microbiome demonstrates dysbiosis in PE patients, and mechanistic studies in animal models suggest that microbiota-derived metabolites may influence placental development through epigenetic pathways; however, clinical evidence in humans remains preliminary. Integration of environmental exposure assessment with multi-omics profiling in large prospective studies is necessary to establish whether environmental factors causally contribute to PE pathogenesis. Future research combining detailed environmental characterization, longitudinal epigenomic profiling, and rigorous causal inference methods will be essential to translate these mechanistic insights into prevention and therapeutic strategies.

## Graphical Abstract

**Graphical Abstract:**
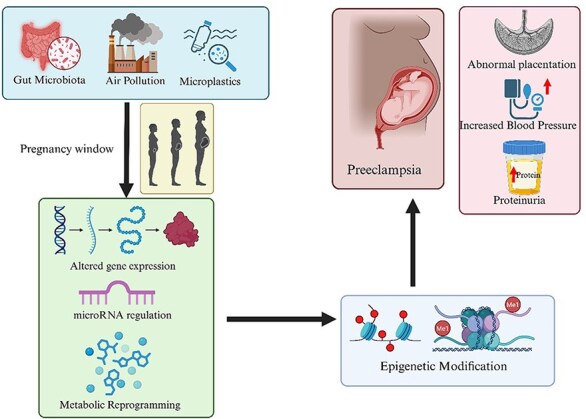

## Introduction

Preeclampsia (PE) is a complex, multisystem hypertensive disorder of pregnancy that affects approximately 2%–8% of pregnancies worldwide and remains a major cause of maternal and perinatal morbidity as well as mortality [1]. Clinically, it is defined by new-onset hypertension, systolic blood pressure ≥140 mmHg or diastolic blood pressure ≥90 mmHg, after 20 weeks gestation, often accompanied by proteinuria or evidence of end-organ dysfunction, such as renal insufficiency, hepatic involvement, neurological complications, or hematological abnormalities [2, 3]. Despite advances in obstetric care, PE accounts for an estimated 46 000 maternal deaths and up to half a million stillbirths and neonatal deaths each year, with disproportionate impact in low- and middle-income countries where diagnostic and treatment resources are limited [4].

The classical “two-stage model” of PE pathophysiology posits that an initial placentation defect, characterized by inadequate trophoblast invasion and shallow spiral artery remodeling, leads to placental hypoxia and oxidative stress, which in turn trigger the maternal clinical syndrome through systemic endothelial activation and inflammation [5]. While this framework has provided a basis for understanding disease mechanisms, it fails to capture the full heterogeneity of PE, including variation in timing (early-onset vs. late-onset), severity, and associated comorbidities such as cardiovascular disease and metabolic syndrome later in life [6]. Additionally, predictive biomarkers and preventive interventions based on angiogenic factors (e.g. sFlt-1 and PlGF), low-dose aspirin, or antioxidant supplementation have resulted in only slight enhancements in outcomes. This reinforces the necessity for better understanding of upstream drivers and new therapeutic targets [7].

Geographic and temporal differences in PE rates worldwide, suggest that genetics alone do not explain disease distribution. Factors associated with modern living, including urban air quality, dietary shifts, and changes in maternal microbial communities, show potential connections to PE but have received limited research attention in understanding PE development [8, 9]. Recent technological improvements in measuring epigenetic marks have revealed that environmental stressors cause detectable changes in DNA methylation, histone proteins, and RNA molecules that persist in placental and maternal tissues, with long-term effects on gene activity [10, 11]. These findings support a framework called “environmental epigenomics”, combining epidemiological investigation with epigenetic analysis, to clarify how exposures before and early in pregnancy influence pregnancy complications through molecular mechanisms. Emerging data highlight two environmental domains of relevance to PE: ambient air pollution and gut microbiome dysbiosis. Fine particulate matter (PM₂.₅) and gaseous pollutants such as NO₂ and O₃ have been robustly linked to increased PE risk in multiple cohorts, with exposure during preconception and the first trimester exerting the strongest effects on early-onset disease [12–14]. Mechanistically, these pollutants provoke oxidative stress, endothelial dysfunction, and inflammatory signaling, which converge on placental vasculature and maternal cardiovascular adaptation. In parallel, maternal gut microbiome analyses reveal marked dysbiosis in PE patients, characterized by reduced diversity and shifts in key taxa that regulate immune tolerance, metabolic homeostasis, and epigenetic cross-talk via microbial metabolites [8, 15]. Animal models confirm that microbiome perturbation impairs placental development and uterine natural killer (NK) cell function, leading to fetal resorption and hypertensive features reminiscent of PE [16].

Taken together, the intersection of environmental exposures and epigenetic mechanisms offers a novel lens through which to understand PE heterogeneity and to identify new preventive and therapeutic strategies. By delineating critical windows of vulnerability, from preconception through early gestation, and mapping the epigenetic signatures induced by air pollution and microbiome alterations, researchers can develop precision medicine approaches that target both environmental risk reduction and epigenetic modulation. This review therefore aims to synthesize current evidence on environmental epigenomics in PE, illuminate key mechanistic pathways, and chart future directions for research and clinical translation.

## Environmental exposures and PE: current evidence

### Air pollution as a novel risk factor

Air pollution exposure during pregnancy has emerged as a significant environmental risk factor for PE development. Recent meta-analyses demonstrate that ambient particulate matter and gaseous air pollution are associated with increased odds of PE, with effect sizes varying by trimester of exposure and pollutant type [9, 13]. The relationship between air pollution and PE is particularly compelling given the mechanistic parallels between air pollution-induced cardiovascular disease and PE pathophysiology including: **oxidative stress and mitochondrial dysfunction**, with PM_2.5_ exposure inducing cytochrome P450/CYP1A1 pathway activation leading to reactive oxygen species (ROS) generation and placental trophoblast damage [17]; **systemic endothelial dysfunction**, characterized by reduced nitric oxide (NO) bioavailability, increased vasoconstriction, and upregulated adhesion molecules (VCAM-1, ICAM-1) [9, 18]; **chronic inflammatory activation**, evidenced by elevated C-reactive protein, interleukin-6 (IL-6), and tumor necrosis factor-alpha (TNF-α) [19–21]; and **angiogenic imbalance**, with air pollution exposure associated with increased sFlt-1/PlGF ratio, a hallmark biomarker of PE, as demonstrated in the LIFECODES prospective birth cohort where PM_2.5_ and NO_2_ exposure during pregnancy significantly elevated sFlt-1/PlGF ratios and reduced free PlGF levels [22].

Fine particulate matter (PM_2.5_) exposure shows the strongest associations with PE risk. A large retrospective cohort study in China involving 116 042 pregnant women found that PM_2.5_, PM_10_, NO_2_, and O_3_ exposures were significant risk factors for PE in the first and second trimesters, with PM_2.5_ showing dose-response relationships [14]. Similarly, a spatiotemporal analysis in Barcelona demonstrated that first- and third-trimester PM_2.5_ exposures were significantly associated with PE development, with stronger associations observed for early-onset PE during first-trimester exposure [9].

The critical exposure windows for air pollution effects on PE appear to be trimester-specific and pollutant-dependent. A recent study in Los Angeles identified a susceptible window from one week preconception to 11 weeks post conception for PM_2.5_ exposure and gestational hypertension development [12]. Notably, women with probable prenatal depression showed increased vulnerability to air pollution exposure, suggesting that psychosocial stress may potentiate environmental effects through epigenetic mechanisms [12].

Source-specific analyses of air pollution exposure distinguish between traffic-related and wood smoke pollutants, revealing markedly different associations with PE subtypes that depend critically on both the timing of exposure during pregnancy and the PE phenotype (early- vs. late-onset), with key findings summarized in Table 1.

**Table 1:** Source-specific air pollutant associations with preeclampsia subtypes.

Pollutant source	Exposure unit	Exposure timing	PE type affected	Effect size and 95% CI	Percentage increase in PE risk
Traffic-related (PM_2.5_) [72]	1.35 µg/m³	Entire pregnancy	PE (all types)	1.26–1.59	42%
Traffic-related (NO_*x*_) [72]	5.65 ppb	Entire pregnancy	PE (all types)	1.18–1.49	33%
		First gestational month (weeks 1–4)	Early-onset PE (<34 weeks)	1.08–1.68	35%
Wood smoke (PM₂.₅) 3.64 µg/m³ [42]		Second gestational month (weeks 5–8)	Early-onset PE (<34 weeks)	1.23–1.86	51%
	Third gestational month (weeks 9–12)	Early-onset PE (<34 weeks)	1.06–1.46	25%	

This stage-specific and source-specific pattern indicates that different pollutant components trigger distinct pathophysiological pathways through differential epigenetic effects on placental development and maternal vascular adaptation like, traffic-related pollution (black carbon, polycyclic aromatic hydrocarbons) induces primarily DNA hypomethylation in placental tissue [23], particularly affecting genes involved in angiogenesis (VEGFA, FLT1) [24], trophoblast invasion (ADORA2B) [25], and inflammatory responses (CYP2E1, RNF39) [26]. In contrast, wood smoke constituents appear to preferentially induce oxidative stress-mediated epigenetic changes, including increased 3-nitrotyrosine formation in placental tissue [27, 28], and, altered histone acetylation patterns (H3K4me2, H3K9ac) [29].

### Gut microbiome dysbiosis and PE

Pregnancy causes major shifts in maternal gut bacteria composition, which normally help support both fetal growth and maternal metabolism [8, 15]. Current research suggests dysbiosis (abnormal bacterial patterns) accompanies PE development and likely plays a role in PE through altered immune function, metabolic imbalance, and epigenetic signaling [8, 15, 30].

Women with PE demonstrate different gut bacterial patterns than those without hypertension. Studies document lower bacterial diversity, higher levels of potentially harmful bacteria (Fusobacterium, Veillonella), and lower levels of beneficial organisms (Faecalibacterium, Akkermansia) in women with PE. These microbial imbalances correlate with blood pressure levels and markers of renal insufficiency, suggesting direct involvement in PE pathophysiology [15].

The temporal dynamics of microbiome changes in PE are particularly revealing. Microbiome alterations are detectable early in pregnancy and persist through 6 weeks postpartum, indicating that dysbiosis may be both a cause and consequence of PE [8]. Longitudinal studies demonstrate that specific microbial metabolites, including trimethylamine-N-oxide (TMAO) and lipopolysaccharide (LPS), are elevated in PE patients and show gestational age-specific associations with PE risk [8].

Recent experimental evidence provides mechanistic insights into microbiome-PE interactions. A groundbreaking study using antibiotic-induced dysbiosis in pregnant mice demonstrated that gut microbiome disruption leads to increased fetal resorption, impaired placental development, and altered vascularization [16]. These adverse outcomes were associated with key pathological features of PE, including hypoxia, endoplasmic reticulum stress, and reduction in uterine NK cell numbers. Importantly, glucose supplementation restored placental NK cell function and reduced fetal resorption, suggesting that microbiome effects on PE are mediated through metabolic reprogramming [16].

## Epigenetic mechanisms in environmental PE programming

The major epigenetic modifications induced by environmental exposures in PE are summarized in Table 2, which highlights the specific environmental triggers and functional consequences of each epigenetic mark.

**Table 2:** Representative epigenetic alterations in PE.

Epigenetic mark	Examples (Genes/miRNAs)	Environmental trigger	Functional consequence
DNA methylation	VEGFA, FLT1, INHA	PM_2.5_, nutrition	Altered angiogenesis/placentation
microRNAs	C19MC, C14MC, miR-1307–3p	Air pollution, psychosocial stress	Dysregulated immune and vascular signaling
Histone modification	Global H3K27ac	Inflammation, diet	Chromatin accessibility changes

### MicroRNA-mediated environmental effects

MicroRNAs represent a critical interface between environmental exposures and gene regulation in PE. Circulating miRNAs and exosomal miRNA signatures exemplify environmental epigenetic programming, with studies demonstrating differential expression in response to air pollution and oxidative stress. Circulating miRNAs in extracellular vesicles serve as both biomarkers and environmental mediators of PE development. Circulating miRNA patterns shift in response to oxidative damage and other environmental factors, making them useful indicators of environmental stress. Exosomal packaged miRNAs offer particularly stable markers that can be tracked across pregnancy, illustrating the chain of events connecting environmental stress to altered miRNA levels to placental problems [10, 11, 31].

Placenta-specific miRNA clusters represent critical interfaces between environmental exposures and gene expression regulation in pregnancy complications [32]. The chromosome 19 microRNA cluster (C19MC), composed of 46 miRNA genes with paternally imprinted expression, demonstrates environmental responsiveness to specific chemical contaminants, including dichlorodiphenyldichloroethylene (DDE), bisphenol A (BPA), polybrominated diphenyl ethers, polychlorinated biphenyls (PCBs), arsenic, mercury, lead, and cadmium, which trigger altered expression of C19MC members miR-517a, miR-517c, miR-522, and miR-23a, among the most highly expressed C19MC miRNAs in placental tissue [33]. These C19MC miRNAs regulate critical placental functions, including trophoblast invasion and extravillous trophoblast differentiation through miR-517a and miR-517c expression, angiogenic signaling through vascular development pathways via miR-522, and immune tolerance at the maternal-fetal interface through miR-23a dysregulation [33, 34]. In contrast, the chromosome 14 microRNA cluster (C14MC), composed of 52 miRNA genes with maternally imprinted expression, regulates trophoblast invasion and anti-inflammatory responses through members such as miR-145 and miR-483 [34]; however, the current literature lacks established associations between specific environmental chemical exposures and C14MC dysregulation, representing a significant research gap where systematic studies examining environmental exposure-induced C14MC alterations in pregnancy have not been conducted.

Prospective investigation identified 148 differentially expressed circulating miRNAs in women who developed PE (13 enriched, 135 reduced). First-trimester dysregulation of pro-inflammatory miRNAs (miR-20a and miR-21) correlates with early-onset PE, while second-trimester downregulation of anti-inflammatory miRNAs (miR-146a and miR-222) amplifies systemic inflammation. Peak vulnerability for environmental miRNA programming occurs periconception to 11 weeks, with enhanced sensitivity during critical placentation windows (6–18 weeks spiral artery remodeling, 1–12 weeks trophoblast invasion). Multiple machine learning algorithms (logistic regression with elastic-net, random forest, support vector machines) with leave-one-out cross-validation achieved AUROC 0.956 (∼90% true positive rate). Top predictive miRNAs (miR-1307–3p, miR-520a-5p) regulate vascular remodeling, cellular proliferation, and angiogenesis, processes disrupted in PE pathophysiology [11].

### DNA methylation and environmental programming

DNA methylation represents a stable epigenetic mechanism through which environmental exposures can induce lasting changes in gene expression patterns [35]. In PE, methylation changes affect key regulatory pathways, including angiogenesis, immune tolerance, and placental development [36]. Environmental factors, particularly air pollution and nutritional factors, can alter DNA methylation patterns during critical windows of pregnancy [37].

Several studies have identified specific CpG sites that show differential methylation in PE placentas compared to controls [38]. These methylation changes affect genes involved in trophoblast invasion (including STOX1 and INHA) [39], angiogenic signaling (VEGFA and FLT1) [40], and immune regulation (HLA-G and IDO1) [41]. Importantly, many of these methylation changes are detectable in maternal blood, suggesting potential for non-invasive biomarker development [37].

Air pollution exposure during pregnancy, particularly fine particulate matter (PM₂.₅), has been shown to induce genome-wide methylation changes in placental tissue [35]. PM₂.₅ exposure specifically alters methylation patterns in genes involved in oxidative stress response, inflammation, and cardiovascular development [35]. Traffic-related pollution and black carbon exposure specifically induce DNA hypomethylation in genes regulating vascular development, while wood smoke exposure during early pregnancy induces alterations in methylation patterns associated with nitrosative stress and oxidative damage [42]. These air pollution-induced methylation changes program PE susceptibility by altering placental vascular development and maternal vascular adaptation to pregnancy [35]. Additionally, nutritional factors, including folate, vitamin B12, and choline status influence DNA methylation capacity and may modify the epigenetic response to environmental exposures during pregnancy [43].

## Metabolic reprogramming through environmental exposures

Environmental factors directly alter the composition of the maternal gut microbiota and the production of microbial metabolites, which in turn can affect placental development and maternal health during pregnancy [8]. High-fat, low-fiber diets reduce the production of SCFAs [44]. In women with PE, reductions in SCFA-producing bacteria have been documented, including Coprococcus [45], Faecalibacterium, and Roseburia [46]. This dietary pattern is accompanied by decreased levels of fecal butyrate and propionate in women with PE [47]. While animal studies have demonstrated that butyrate supplementation reduces blood pressure and inflammatory markers in pregnancy models, the direct causal mechanism in human PE requires further clinical validation [47].

Air pollution exposure represents an emerging environmental factor affecting the maternal microbiome. Recent evidence suggests that air pollution exposure, including particulate matter, may contribute to dysbiosis during pregnancy through mechanisms involving oxidative stress and inflammatory pathways [30]. Dysbiotic microbiota alterations in PE are characterized by increased relative abundance of Proteobacteria and reduced Firmicutes, bacterial phyla that include both pathogenic species and SCFA-producing bacteria, though the specific mechanisms by which air pollution directly alters these bacterial populations in pregnant women remain incompletely understood [30].

**Figure 1:**
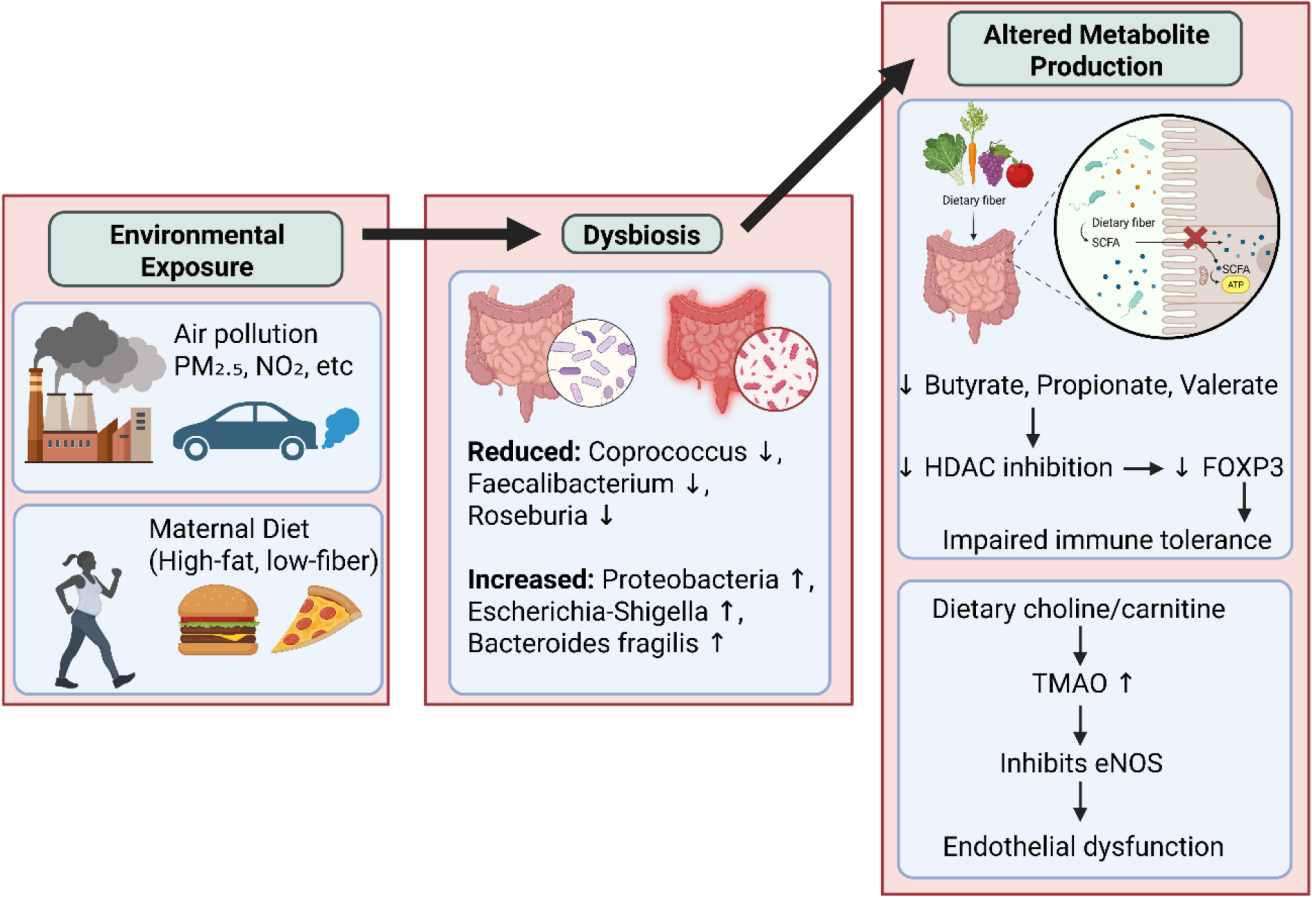
Environmental exposures drive metabolic reprogramming through dysbiosis-mediated alterations in microbial metabolite production.

Production of metabolites by the dysbiotic microbiota contributes to PE pathogenesis through multiple pathways [48]. Dysbiosis reduces the capacity of the microbiota to produce adequate SCFA levels, and these metabolites regulate immune homeostasis through histone deacetylase (HDAC) inhibition and epigenetic regulation of genes involved in immune tolerance and vascular development shown in Fig. 1 [49]. Recent studies report mixed evidence on Trimethylamine-N-oxide (TMAO) and PE a risk. While some clinical cohorts found that TMAO and its precursors are not associated with increased PE risk in maternal blood [50], other studies report that TMAO levels are elevated at delivery in early and severe PE, suggesting a possible role in disease progression rather than initiation [51].

Bile acid metabolism is also altered in PE and may be influenced by microbiota composition. Changes in bacterial bile salt hydrolase activity and alterations in the relative abundance of Bacteroidetes have been associated with modified bile acid profiles in pregnancy complications [52]. The altered bile acid metabolism may affect farnesoid X receptor (FXR) signaling, which is involved in the regulation of metabolic and immune homeostasis [52]. While epigenetic modifications of genes involved in these pathways have been documented in PE, direct evidence linking specific environmental exposures to dysbiosis-mediated epigenetic changes in the placenta in humans remains limited and needs further investigation [53].

## Critical windows of environmental susceptibility

Environmental vulnerability during pregnancy is temporally dynamic, with distinct critical periods of heightened susceptibility to specific exposures (Table 3). Understanding these temporal patterns is crucial for identifying optimal windows for intervention and predicting which environmental exposures will exert maximal effects on PE risk.

**Table 3:** Gestational windows, vulnerable processes, and environmental risks.

Period	Key processes	Environmental exposures	Mechanisms of injury
Preconception	Oocyte quality, early placentation	PM_2.5_, diet, stress	Epigenetic reprogramming
1st trimester	Trophoblast invasion	Air pollution, microbiome dysbiosis	Impaired spiral artery remodeling
2nd/3rd trimester	Vascular adaptation	Cumulative exposures	Systemic inflammation, oxidative stress

### Preconceptional programming

The preconceptional period represents a critical window for environmental programming of PE susceptibility. Environmental exposures during this period can influence oocyte quality, early embryonic development, and initial placentation processes [54]. Recent evidence suggests that air pollution exposure during the periconceptional period (8 weeks before to 10 weeks after conception) is associated with increased PE risk, particularly in women with psychosocial stress [12].

Preconceptional nutritional status and microbiome composition may establish baseline conditions that influence subsequent pregnancy outcomes. Women with pre-existing microbiome dysbiosis may be more susceptible to PE development, suggesting that preconceptional interventions could modify disease risk [8].

### First trimester: placentation and early programming

The first trimester represents the most critical window for environmental programming of PE, coinciding with key events in placental development, including trophoblast invasion, spiral artery remodeling, and establishment of maternal-fetal immune tolerance [54]. Environmental exposures during this period can disrupt these processes through epigenetic mechanisms.

Air pollution exposure during the first trimester shows the strongest associations with early-onset PE, suggesting that environmental disruption of early placentation processes may have lasting consequences for pregnancy outcomes [9, 13]. The timing of these associations aligns with the critical period for spiral artery remodeling (6–18 weeks of gestation), supporting the proposed mechanism whereby environmental exposures reprogram placental epigenetics during early pregnancy, though direct causal evidence requires further investigation.

Microbiome composition during the first trimester is particularly important for establishing immune tolerance and metabolic adaptation to pregnancy [8]. Dysbiosis during this period may program inflammatory responses and metabolic dysfunction that contribute to PE development later in pregnancy.

### Second and third trimester: amplification of environmental effects

While the first trimester appears most critical for PE programming, second and third trimester exposures can amplify existing susceptibilities or trigger PE in previously programmed individuals. Late pregnancy environmental exposures may be particularly important for late-onset PE, which has distinct pathophysiological characteristics from early-onset disease [9, 13].

The differential effects of environmental exposures across pregnancy trimesters suggest that PE may represent a spectrum of environmentally programmed diseases with distinct etiologies depending on the timing and nature of exposures.

## Mechanistic pathways linking environment and PE

### Oxidative stress and mitochondrial dysfunction

Environmental exposures, particularly air pollution, induce oxidative stress that may trigger cascading pathophysiological changes leading to PE [55, 56]. Fine particulate matter and gaseous pollutants generate ROS that overwhelm cellular antioxidant defenses and induce mitochondrial dysfunction [55, 56].

Mitochondrial dysfunction is emerging as a central mechanism in PE pathogenesis, with evidence of impaired oxidative phosphorylation, increased mitochondrial ROS production, and altered mitochondrial biogenesis in PE placentas [55]. Environmental pollutants may directly damage mitochondrial DNA and alter mitochondrial function through epigenetic mechanisms affecting mitochondrial gene expression.

The connection between environmental oxidative stress and PE is further supported by evidence that mitochondrial-targeted antioxidants may ameliorate PE-like symptoms in animal models [57]. However, translation of these preclinical findings to clinical prevention of PE in humans remains unproven and requires prospective human trials.

### Inflammatory programming

Environmental exposures induce chronic low-grade inflammation that may program PE susceptibility through effects on immune tolerance and vascular function [8, 13]. Air pollution exposure activates inflammatory pathways, including NF-κB signaling, which may alter gene expression patterns relevant to placental development and maternal vascular adaptation.

Microbiome dysbiosis contributes to inflammatory programming through increased production of pro-inflammatory metabolites and reduced production of anti-inflammatory factors [8]. The resulting inflammatory milieu may alter immune tolerance at the maternal-fetal interface and contribute to the inflammatory component of PE pathophysiology.

### Vascular programming

Environmental exposures may program vascular dysfunction that predisposes to PE through effects on endothelial function, vascular remodeling, and blood pressure regulation [9, 13]. Air pollution exposure is associated with endothelial dysfunction and increased cardiovascular disease risk in non-pregnant populations, suggesting similar mechanisms may operate during pregnancy.

**Figure 2:**
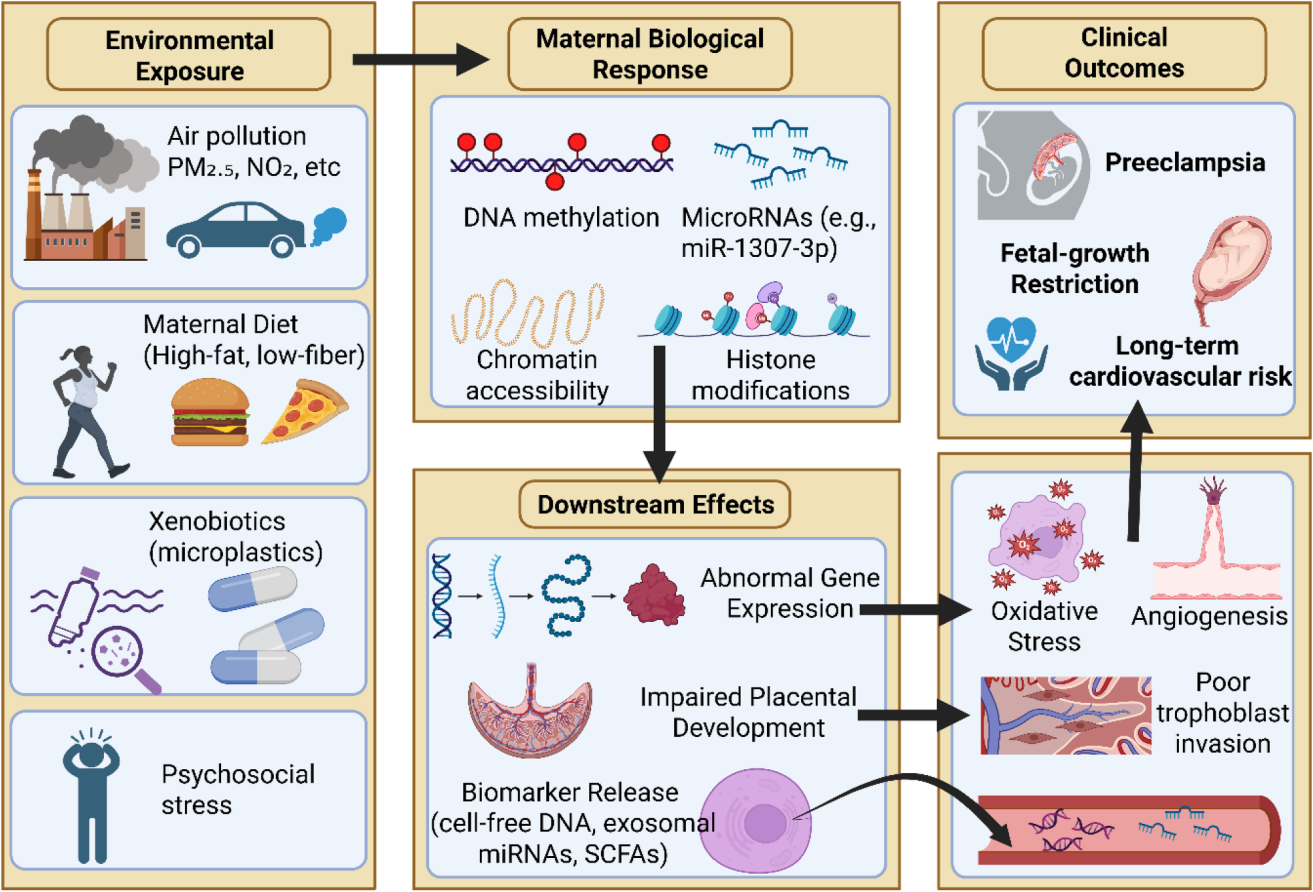
Environmental exposures induce epigenetic alterations and downstream biological responses leading to PE.

The gut microbiome influences vascular function through production of vasoactive metabolites and regulation of NO signaling [8]. Microbiome dysbiosis may contribute to the vascular dysfunction observed in PE through altered production of these regulatory factors. The interconnected pathways linking specific environmental exposures to PE via epigenetic modifications are depicted in Fig. 2.

## Precision medicine approaches in environmental PE

### Environmental risk stratification

Understanding environmental contributions to PE enables development of precision medicine approaches that incorporate environmental risk factors into clinical decision-making [58]. While current machine learning models for PE risk prediction primarily integrate clinical, genetic, and laboratory variables, emerging approaches demonstrate the potential for systematic environmental variable integration to enhance predictive accuracy [59]. Recent studies demonstrate that machine learning algorithms combining multiple data types, including clinical variables from electronic health records, biochemical biomarkers (such as sFlt-1/PlGF ratios), and demographic information, achieve area under the curve values ranging from 0.86 to 0.97 [60, 61]. However, systematic integration of directly measured environmental variables, including ambient air pollution metrics (PM₂.₅, NO₂), dietary composition data, and microbiome dysbiosis markers, remains limited in current clinical applications.

To fully leverage environmental epigenomics insights for precision medicine, future machine learning models should prospectively integrate measured environmental exposures. This integration could involve: (1) air pollution metrics such as PM₂.₅ and NO₂ levels from regulatory monitoring networks or personal exposure monitors; (2) microbiome composition data from maternal fecal sampling with quantified dysbiosis indices; (3) dietary patterns characterized by macronutrient and fiber intake; and (4) metabolite profiles including circulating short-chain fatty acids and TMAO levels.

Environmental risk stratification may be particularly valuable for identifying high-risk populations in urban areas with high air pollution exposure or in regions with specific environmental hazards. Personalized environmental exposure assessment could inform targeted interventions and monitoring strategies.

Recent studies now implicate microplastics, ubiquitous environmental contaminants, in the pathogenesis of PE. Microplastics can cross the placental barrier, accumulate in fetal and maternal tissues, and provoke oxidative stress and inflammatory signaling within the placenta. A holistic review highlights how microplastic exposure during pregnancy disrupts endocrine function, induces systemic inflammation, and perturbs placental nutrient transport, thereby creating a “double‐hit” scenario of maternal–fetal stress that mirrors PE risk factors [62]. Complementing these epidemiological insights, in vitro and in vivo data demonstrate that microplastic particles trigger trophoblast ferroptosis via HIF-1α/TFRC upregulation, leading to diminished syncytialization, impaired spiral artery remodeling, and hypertension in pregnant models, hallmarks of PE pathophysiology [63]. Together, these findings establish microplastics as a novel environmental epigenomic insulter that may exacerbate placental dysfunction and maternal vascular maladaptation in PE.

### Biomarker development

Environmental epigenomics offers novel opportunities for biomarker development in PE through identification of epigenetic signatures that reflect both disease status and environmental exposure history. Circulating microRNAs packaged in extracellular vesicles represent particularly promising environmental biomarkers, as demonstrated by a prospective study, identifying 148 differentially expressed miRNAs in women with PE, with the most predictive miRNAs, hsa-miR-1307-3p and hsa-miR-520a-5p, being responsive to oxidative stress and inflammatory stimuli associated with air pollution exposure (PM₂.₅ and NO₂) and achieving an area under the receiver operating characteristic curve of 0.956 for early disease detection [11].

Differential DNA methylation patterns in maternal blood and placental tissue provide stable epigenetic signatures directly correlating with environmental exposure history, with specific CpG sites in placental development genes (VEGFA, FLT1, STOX1, INHA) showing differential methylation in women exposed to high-level PM₂.₅ during early pregnancy [39–41], while dietary exposures, particularly high-fat, low-fiber diets altering the gut microbiome, correlate with specific DNA methylation patterns in immune regulation gene (FOXP3) measured in circulating cell-free DNA [44].

Placental exosomes serve as particularly rich sources of environmental biomarkers, carrying molecular cargo directly reflecting placental response to environmental insults, with exosomal markers of oxidative stress and inflammation showing dose-dependent correlations with measured maternal PM₂.₅ exposure levels [10].

Circulating microbiome-derived metabolites, particularly short-chain fatty acids (butyrate, propionate, and, valerate), serve as biomarkers of dysbiosis induced by environmental dietary pattern changes, with maternal fecal or serum SCFA concentrations measured at 10–15 weeks of gestation significantly predicting PE development [45, 47, 64].

### Targeted interventions

Environmental epigenomics insights enable development of targeted interventions that address specific environmental risk factors or their downstream effects [58, 65]. These may include environmental modification strategies, nutritional interventions, or pharmacological approaches targeting environmental pathways.

Microbiome-targeted interventions, including probiotics, prebiotics, and dietary modifications, represent promising approaches for PE prevention [8, 15]. Clinical trials of these interventions should be designed based on mechanistic understanding of microbiome-PE interactions and individual risk stratification.

Antioxidant interventions targeting environmental oxidative stress may be effective for PE prevention, particularly mitochondrial-targeted compounds that address the cellular consequences of environmental exposures [56, 66]. The timing and targeting of these interventions should be informed by understanding of critical exposure windows and individual susceptibility factors.

## Clinical implications and future directions

### Redefining PE prevention

Environmental epigenomics insights necessitate a fundamental shift in PE prevention strategies from generic interventions to personalized approaches based on environmental risk assessment [65]. Future prevention programs should incorporate environmental exposure assessment, genetic susceptibility screening, and targeted interventions based on individual risk profiles.

Preconceptional counselling should include environmental risk assessment and modification strategies, particularly for women living in high-pollution areas or with other environmental risk factors [12]. Public health interventions targeting air quality improvement and environmental health promotion may have significant impacts on PE incidence at the population level.

### Clinical practice integration

Integration of environmental risk factors into clinical practice requires development of practical assessment tools and decision-support systems [67, 68]. Electronic health records should incorporate environmental exposure data, and clinical algorithms should be updated to include environmental risk factors alongside traditional clinical predictors.

Healthcare providers need education and training on environmental health risks during pregnancy and strategies for risk modification. Development of clinical guidelines that address environmental factors in PE risk assessment and management will be essential for widespread implementation.

## Research priorities

Future research should focus on several key areas to advance understanding of environmental contributions to PE:

- Large-scale longitudinal studies that combine detailed environmental exposure assessment with multi-omics profiling to identify causal relationships and critical exposure windows [13, 54].
- Mechanistic studies using in vitro and animal models to understand cellular and molecular pathways through which environmental exposures program PE susceptibility [16, 69].
- Intervention trials testing environmental modification strategies, including air pollution reduction, microbiome interventions, and targeted nutritional approaches [8, 70].
- Biomarker validation studies to translate environmental epigenomics discoveries into clinically useful predictive tests [11, 66].
- Health disparities research to understand how environmental inequities contribute to disparities in PE outcomes and develop targeted interventions for high-risk populations [12].

## Challenges and limitations

### Methodological challenges

Environmental epigenomics research in PE faces several methodological challenges that must be addressed to advance the field. Environmental exposure assessment is complex and requires sophisticated methods to capture temporal variations, mixture effects, and individual-level exposures [9, 13]. Personal exposure monitoring, biomarker-based exposure assessment, and advanced exposure modeling approaches will be necessary to improve exposure characterization.

Epigenetic analyses require standardized protocols for sample collection, processing, and analysis to ensure reproducibility across studies [10, 11]. The temporal dynamics of epigenetic changes during pregnancy add complexity to study design and interpretation. Integration of multi-omics data (genomics, transcriptomics, epigenomics, metabolomics) requires advanced bioinformatics approaches and standardized analytical pipelines.

### Causal inference

Establishing causal links between environmental exposures and preeclampsia remains difficult, largely because most available studies are observational and involve a complex mixture of interacting factors [13]. Mendelian randomization, which uses genetic variants as proxies for environmental exposures, offers a promising approach to strengthen causal inference. However, it requires very large datasets and careful selection of valid genetic instruments to ensure reliable results.

Experimental studies in animal models have helped uncover potential mechanisms, but they cannot fully replicate the unique features of human PE [71]. Advances in human-based model systems, such as placental organoids and organ-on-chip technologies, hold promise for bridging this gap by providing more physiologically relevant platforms for studying how environmental factors affect placental biology and PE development.

### Clinical translation

Translating environmental epigenomics findings into clinical practice comes with several challenges. These include the complexity of accurately assessing environmental risk, the need for specialized laboratory infrastructure, and the difficulty of integrating new tools into existing clinical workflows [67]. Cost-effectiveness studies will also be important to determine whether implementing environmental risk assessments and targeted interventions is feasible and sustainable.

Regulatory pathways for environmental biomarkers and intervention strategies may differ from those used for traditional pharmaceuticals, making it necessary to establish new validation and approval frameworks [68]. Close collaboration between researchers, clinicians, regulatory bodies, and public health authorities will be crucial to ensure that these advances can be safely and effectively translated into clinical care.

## Conclusion

Environmental epigenomics is reshaping how we understand the development of PE, showing that exposures during key stages of pregnancy can leave lasting epigenetic marks that influence disease risk. Growing evidence, especially around air pollution and gut microbiome alterations, highlights how strongly environmental factors contribute to this complex condition.

Bringing these environmental influences into both research and clinical practice opens new doors for prevention, earlier detection, and more personalized care. When environmental risk profiling is combined with genomic and clinical information, it has the potential to support true precision medicine for improving outcomes in mothers and their babies.

At the same time, there are still major challenges to overcome, particularly in refining research methods, proving causal links, and translating findings into real-world clinical use. Moving forward will require close collaboration among researchers, clinicians, and public health teams to build the evidence, tools, and systems needed to make these advances a reality.

Future research should focus on large, long-term studies that pair detailed environmental exposure assessments with multi-omics data, along with mechanistic studies that clarify how these exposures influence biological pathways leading to disease. Intervention trials that explore whether modifying environmental risk factors can reduce the incidence or severity of PE will also be essential. Together, these efforts aim to build a comprehensive picture of how environmental influences contribute to PE and to support prevention and treatment strategies tailored to a woman’s individual risk and exposure profile.

The idea of environmental epigenomics in PE is more than just a new research direction, it provides a powerful lens for understanding how modern environmental pressures intersect with maternal health during one of the most sensitive phases of life. As challenges like climate change, rapid urbanization, and rising pollution continue to intensify, uncovering these connections becomes increasingly important for safeguarding the health of mothers and their babies.

## References

[1] Jeyabalan A. Epidemiology of preeclampsia: impact of obesity. Nutr Rev. 2013;71:S18–25. 24147919 10.1111/nure.12055 PMC3871181

[2] Rana S, Lemoine E, Granger JP et al. Preeclampsia. Circ Res. 2019;124:1094–112. 10.1161/CIRCRESAHA.118.313276 30920918

[3] Vest AR, Cho LS. Hypertension in pregnancy. Curr Atheroscler Rep. 2014;16:395. 10.1007/s11883-013-0395-8 24477794

[4] Cresswell JA, Alexander M, Chong MYC et al. Global and regional causes of maternal deaths 2009–20: a WHO systematic analysis. Lancet Glob Heal. 2025;13:e626–34. https://linkinghub.elsevier.com/retrieve/pii/S2214109×24005606 10.1016/S2214-109X(24)00560-6 PMC11946934 40064189

[5] Tomimatsu T, Mimura K, Endo M et al. Pathophysiology of preeclampsia: an angiogenic imbalance and long-lasting systemic vascular dysfunction. Hypertens Res. 2017;40:305–10. https://www.nature.com/articles/hr2016152 27829661 10.1038/hr.2016.152

[6] Suvakov S, Vaughan LE, Parashuram S et al. Women with a history of preeclampsia exhibit accelerated aging and unfavorable profiles of senescence markers. Hypertension. 2024;81:1550–60. 10.1161/HYPERTENSIONAHA.123.22250 38690656 PMC11168873

[7] Dickerson AG, Joseph CA, Kashfi K. Current approaches and innovations in managing preeclampsia: highlighting maternal health disparities. J Clin Med. 2025;14:1190. https://www.mdpi.com/2077-0383/14/4/1190 40004721 10.3390/jcm14041190 PMC11856135

[8] Ishimwe JA. Maternal microbiome in preeclampsia pathophysiology and implications on offspring health. Physiol Rep. 2021;9:1–19. 10.14814/phy2.14875 PMC8157769 34042284

[9] Dadvand P, Figueras F, Basagaña X et al. Ambient air pollution and preeclampsia: a spatiotemporal analysis. Environ Health Perspect. 2013;121:1365–71. 10.1289/ehp.1206430 24021707 PMC3855505

[10] Giannubilo SR, Cecati M, Marzioni D et al. Circulating miRNAs and preeclampsia: from implantation to epigenetics. Int J Mol Sci. 2024;25:1418. https://www.mdpi.com/1422-0067/25/3/1418 38338700 10.3390/ijms25031418 PMC10855731

[11] Ghosh S, Thamotharan S, Fong J et al. Circulating extracellular vesicular microRNA signatures in early gestation show an association with subsequent clinical features of pre-eclampsia. Sci Rep. 2024;14:16770. 10.1038/s41598-024-64057-w 39039088 PMC11263608

[12] Niu Z, Habre R, Yang T et al. Increased risk of gestational hypertension by periconceptional exposure to ambient air pollution and effect modification by prenatal depression. Hypertension. 2024;81:1285–95. 10.1161/HYPERTENSIONAHA.123.22272 38533642 PMC11096032

[13] Hu H, Bian J, Zhao J. Ambient air pollution and preeclampsia. Hypertension. 2020;75:618–19. 10.1161/HYPERTENSIONAHA.119.13269 31902251 PMC7035148

[14] Jia L, Liu Q, Hou H et al. Association of Ambient air pollution with risk of preeclampsia during pregnancy: a retrospective cohort study. BMC Public Health. 2020;20:1663. https://bmcpublichealth.biomedcentral.com/articles/10.1186/s12889-020-09719-w 33153479 10.1186/s12889-020-09719-w PMC7643463

[15] Zong Y, Wang X, Wang J. Research progress on the correlation between gut microbiota and preeclampsia: microbiome changes, mechanisms and treatments. Front Cell Infect Microbiol. 2023;13:1–15. https://www.frontiersin.org/articles/10.3389/fcimb.2023.1256940/full 10.3389/fcimb.2023.1256940 PMC10644267 38029244

[16] Giugliano S, Gatti A, Rusin M et al. Maternal gut microbiota influences immune activation at the maternal-fetal interface affecting pregnancy outcome. Nat Commun. 2025;16:4326. https://www.nature.com/articles/s41467-025-58533-8 40346042 10.1038/s41467-025-58533-8 PMC12064790

[17] Li S, Li L, Zhang C et al. PM2.5 leads to adverse pregnancy outcomes by inducing trophoblast oxidative stress and mitochondrial apoptosis via KLF9/CYP1A1 transcriptional axis. Elife. 2023;12:1–35. https://elifesciences.org/articles/85944 10.7554/eLife.85944 PMC10584374 37737576

[18] Sun Y, Bhuyan R, Jiao A et al. Association between particulate air pollution and hypertensive disorders in pregnancy: a retrospective cohort study. PLOS Med. 2024;21:e1004395. 10.1371/journal.pmed.1004395 38669277 PMC11087068

[19] Gogna P, Borghese MM, Villeneuve PJ et al. A cohort study of the multipollutant effects of PM_2.5_, NO_2_, and O_3_ on C-reactive protein levels during pregnancy. Environ Epidemiol. 2024;8:e308. https://journals.lww.com/10.1097/EE9.0000000000000308 38799262 10.1097/EE9.0000000000000308 PMC11115979

[20] Craig EA, Lin Y, Ge Y et al. Associations of gestational exposure to air pollution and polycyclic aromatic hydrocarbons with placental inflammation. Environ Heal. 2024;2:672–80. 10.1021/envhealth.4c00077 PMC11420950 39323894

[21] Friedman C, Dabelea D, Thomas DSK et al. Exposure to ambient air pollution during pregnancy and inflammatory biomarkers in maternal and umbilical cord blood: the healthy start study. Environ Res. 2021;197:111165. https://linkinghub.elsevier.com/retrieve/pii/S001393512100459X 33857458 10.1016/j.envres.2021.111165 PMC8216209

[22] Zheng Y, McElrath T, Cantonwine D et al. Longitudinal associations between ambient air pollution and angiogenic biomarkers among pregnant women in the LIFECODES Study, 2006–2008. Environ Health Perspect. 2023;131:2006–8. 10.1289/EHP11909 PMC10411633 37556304

[23] Isaevska E, Moccia C, Asta F et al. Exposure to ambient air pollution in the first 1000 days of life and alterations in the DNA methylome and telomere length in children: a systematic review. Environ Res. 2021;193:110504. 10.1016/j.envres.2020.110504 33221306

[24] Soto SDF, Melo JOd, Marchesi GD et al. Exposure to fine particulate matter in the air alters placental structure and the renin-angiotensin system. PLoS One. 2017;12:e0183314. https://dx.plos.org/10.1371/journal.pone.0183314 28820906 10.1371/journal.pone.0183314 PMC5562329

[25] Engström K, Mandakh Y, Garmire L et al. Early pregnancy exposure to ambient air pollution among late-onset preeclamptic cases is associated with placental DNA hypomethylation of specific genes and slower placental maturation. Toxics. 2021;9:338. https://www.mdpi.com/2305-6304/9/12/338 34941772 10.3390/toxics9120338 PMC8708250

[26] Ladd-Acosta C, Feinberg JI, Brown SC et al. Epigenetic marks of prenatal air pollution exposure found in multiple tissues relevant for child health. Environ Int. 2019;126:363–76. https://linkinghub.elsevier.com/retrieve/pii/S0160412018322724 30826615 10.1016/j.envint.2019.02.028 PMC6446941

[27] Ruano CSM, Miralles F, Méhats C et al. The impact of oxidative stress of environmental origin on the onset of placental diseases. Antioxidants. 2022;11:106. https://www.mdpi.com/2076-3921/11/1/106 35052610 10.3390/antiox11010106 PMC8773163

[28] Erlandsson L, Lindgren R, Nääv Å et al. Exposure to wood smoke particles leads to inflammation, disrupted proliferation and damage to cellular structures in a human first trimester trophoblast cell line. Environ Pollut. 2020;264:114790. https://linkinghub.elsevier.com/retrieve/pii/S0269749120316055 32417587 10.1016/j.envpol.2020.114790

[29] Jung YS, Aguilera J, Kaushik A et al. Impact of air pollution exposure on cytokines and histone modification profiles at single-cell levels during pregnancy. Sci Adv. 2024;10:1–17. 10.1126/sciadv.adp5227 PMC11606498 39612334

[30] Deady C, McCarthy FP, Barron A et al. An altered gut microbiome in pre-eclampsia: cause or consequence. Front Cell Infect Microbiol. 2024;14:1–12. https://www.frontiersin.org/articles/10.3389/fcimb.2024.1352267/full 10.3389/fcimb.2024.1352267 PMC11106424 38774629

[31] Shan Y, Hou B, Wang J et al. Exploring the role of exosomal MicroRNAs as potential biomarkers in preeclampsia. Front Immunol. 2024;15:1–16. https://www.frontiersin.org/articles/10.3389/fimmu.2024.1385950/full 10.3389/fimmu.2024.1385950 PMC10985148 38566996

[32] Vrijens K, Bollati V, Nawrot TS. MicroRNAs as potential signatures of environmental exposure or effect: a systematic review. Environ Health Perspect. 2015;123:399–411. 10.1289/ehp.1408459 25616258 PMC4421768

[33] Li Q, Kappil MA, Li A et al. Exploring the associations between microRNA expression profiles and environmental pollutants in human placenta from the National Children’s Study (NCS). Epigenetics. 2015;10:793–802. https://doi.org/full/10.1080/15592294.2015.1066960 26252056 10.1080/15592294.2015.1066960 PMC4622837

[34] Addo KA, Palakodety N, Hartwell HJ et al. Placental microRNAs: responders to environmental chemicals and mediators of pathophysiology of the human placenta. Toxicol Reports. 2020;7:1046–56. 10.1016/j.toxrep.2020.08.002 PMC7472806 32913718

[35] Janssen BG, Godderis L, Pieters N et al. Placental DNA hypomethylation in association with particulate air pollution in early life. Part Fibre Toxicol. 2013;10:22. https://particleandfibretoxicology.biomedcentral.com/articles/10.1186/1743-8977-10-22 23742113 10.1186/1743-8977-10-22 PMC3686623

[36] Meng Y, Meng Y, Li L et al. The role of DNA methylation in placental development and its implications for preeclampsia. Front Cell Dev Biol. 2024;12:1–9. https://www.frontiersin.org/articles/10.3389/fcell.2024.1494072/full 10.3389/fcell.2024.1494072 PMC11649665 39691449

[37] Broséus L, Guilbert A, Hough I et al. Placental DNA methylation signatures of prenatal air pollution exposure and potential effects on birth outcomes: an analysis of three prospective cohorts. Lancet Planet Heal. 2024;8:e297–308. https://linkinghub.elsevier.com/retrieve/pii/S2542519624000457 10.1016/S2542-5196(24)00045-7 38723642

[38] Hjort L, Novakovic B, Cvitic S et al. Placental DNA methylation in pregnancies complicated by maternal diabetes and/or obesity: state of the art and research gaps. Epigenetics. 2022;17:2188–208. 10.1080/15592294.2022.2111755 35950598 PMC9665149

[39] Kohan-Ghadr H-R, Kadam L, Jain C et al. Potential role of epigenetic mechanisms in regulation of trophoblast differentiation, migration, and invasion in the human placenta. Cell Adh Migr. 2016;10:126–35. 10.1080/19336918.2015.1098800 26745760 PMC4853046

[40] Sundrani DP, Reddy US, Joshi AA et al. Differential placental methylation and expression of VEGF, FLT- 1 and KDR genes in human term and preterm preeclampsia. Clin Epigenetics. 2013;5:6. https://clinicalepigeneticsjournal.biomedcentral.com/articles/10.1186/1868-7083-5-6 23621880 10.1186/1868-7083-5-6 PMC3640948

[41] Apicella C, Ruano CSM, Méhats C et al. The role of epigenetics in placental development and the etiology of preeclampsia. Int J Mol Sci. 2019;20:2837. https://www.mdpi.com/1422-0067/20/11/2837 31212604 10.3390/ijms20112837 PMC6600551

[42] Assibey-Mensah V, Glantz JC, Hopke PK et al. Wintertime wood smoke, traffic particle pollution, and preeclampsia. Hypertension. 2020;75:851–58. 10.1161/HYPERTENSIONAHA.119.13139 31902253 PMC7035201

[43] Socha MW, Flis W, Wartęga M. Epigenetic genome modifications during pregnancy: the impact of essential nutritional supplements on DNA methylation. Nutrients. 2024;16:678. https://www.mdpi.com/2072-6643/16/5/678 38474806 10.3390/nu16050678 PMC10934520

[44] Ziętek M, Celewicz Z, Szczuko M. Short-chain fatty acids, maternal microbiota and metabolism in pregnancy. Nutrients. 2021;13:1244. https://www.mdpi.com/2072-6643/13/4/1244 33918804 10.3390/nu13041244 PMC8069164

[45] Altemani F, Barrett HL, Gomez-Arango L et al. Pregnant women who develop preeclampsia have lower abundance of the butyrate-producer Coprococcus in their gut microbiota. Pregnancy Hypertens. 2021;23:211–19. https://linkinghub.elsevier.com/retrieve/pii/S2210778921000015 33530034 10.1016/j.preghy.2021.01.002

[46] Liu K, Fu Q. Microbiome of the different body sites in preeclampsia patients to reveal the role of bacteria in the multifactorial causes. SM J Community Med. 2020;5:1–7. https://www.jsmcentral.org/assets/articles/smjcm473782.pdf

[47] Chang Y, Chen Y, Zhou Q et al. Short-chain fatty acids accompanying changes in the gut microbiome contribute to the development of hypertension in patients with preeclampsia. Clin Sci. 2020;134:289–302. https://portlandpress.com/clinsci/article/134/2/289/221919/Short-chain-fatty-acids-accompanying-changes-in 10.1042/CS20191253 31961431

[48] Torres-Torres J, Basurto-Serrano JA, Camacho-Martinez ZA et al. Microbiota dysbiosis: a key modulator in preeclampsia pathogenesis and its therapeutic potential. Microorganisms. 2025;13:245. https://www.mdpi.com/2076-2607/13/2/245 40005611 10.3390/microorganisms13020245 PMC11857279

[49] Du Y, He C, An Y et al. The role of short chain fatty acids in inflammation and body health. Int J Mol Sci. 2024;25:7379. https://www.mdpi.com/1422-0067/25/13/7379 39000498 10.3390/ijms25137379 PMC11242198

[50] McArthur KL, Zhang M, Hong X et al. Trimethylamine N-oxide and its precursors are associated with gestational diabetes mellitus and pre-eclampsia in the Boston Birth Cohort. Curr Dev Nutr. 2022;6:nzac108. 10.1093/cdn/nzac108 35949367 PMC9356535

[51] Huang X, Li Z, Gao Z et al. Association between risk of preeclampsia and maternal plasma trimethylamine-N-oxide in second trimester and at the time of delivery. BMC Pregnancy Childbirth. 2020;20:302. https://bmcpregnancychildbirth.biomedcentral.com/articles/10.1186/s12884-020-02997-7 32429856 10.1186/s12884-020-02997-7 PMC7236207

[52] Tang B, Tang L, Li S et al. Gut microbiota alters host bile acid metabolism to contribute to intrahepatic cholestasis of pregnancy. Nat Commun. 2023;14:1305. https://www.nature.com/articles/s41467-023-36981-4 36894566 10.1038/s41467-023-36981-4 PMC9998625

[53] Wan Y-JY, Sheng L. Regulation of bile acid receptor activity. Liver Res. 2018;2:180–85. 10.1016/j.livres.2018.09.008 32280557 PMC7147511

[54] Maric-Bilkan C, Abrahams VM, Arteaga SS et al. Research recommendations from the National Institutes of Health workshop on predicting, preventing, and treating preeclampsia. Hypertension. 2019;73:757–66. 10.1161/HYPERTENSIONAHA.118.11644 30686084 PMC6416073

[55] Deer E, LaMarca B, Reckelhoff JF et al. The role of mitochondrial dysfunction and oxidative stress in women’s reproductive disorders: implications for polycystic ovary syndrome and preeclampsia. Int J Mol Sci. 2025;26:6439. https://www.mdpi.com/1422-0067/26/13/6439 40650215 10.3390/ijms26136439 PMC12249776

[56] Chen X, Zhong R, Hu B. Mitochondrial dysfunction in the pathogenesis of acute pancreatitis. Hepatobiliary Pancreat Dis Int. 2025;24:76–83. https://linkinghub.elsevier.com/retrieve/pii/S1499387223002461 38212158 10.1016/j.hbpd.2023.12.008

[57] Opichka MA, Livergood MC, Balapattabi K et al. Mitochondrial-targeted antioxidant attenuates preeclampsia-like phenotypes induced by syncytiotrophoblast-specific Gαq signaling. Sci Adv. 2023;9:1–19. 10.1126/sciadv.adg8118 PMC10691776 38039359

[58] Ashraf UM, Hall DL, Rawls AZ et al. Epigenetic processes during preeclampsia and effects on fetal development and chronic health. Clin Sci. 2021;135:2307–27. https://portlandpress.com/clinsci/article/135/19/2307/229932/Epigenetic-processes-during-preeclampsia-and 10.1042/CS20190070 PMC8948502 34643675

[59] Bhosale YH, Nakkella M, Rani SBE et al. AI-enabled early detection of preeclampsia: a predictive model based on multivariate biomarker analysis. Vasc Endovasc Rev. 2025;8:296–303. https://verjournal.com/index.php/ver/article/view/173

[60] Li T, Xu M, Wang Y et al. Prediction model of preeclampsia using machine learning based methods: a population based cohort study in China. Front Endocrinol (Lausanne). 2024;15:1–14. https://www.frontiersin.org/articles/10.3389/fendo.2024.1345573/full 10.3389/fendo.2024.1345573 PMC11198873 38919479

[61] Butler L, Gunturkun F, Chinthala L et al. AI-based preeclampsia detection and prediction with electrocardiogram data. Front Cardiovasc Med. 2024;11:1–8. https://www.frontiersin.org/articles/10.3389/fcvm.2024.1360238/full 10.3389/fcvm.2024.1360238 PMC10945012 38500752

[62] Paul I, Mondal P, Haldar D et al. Beyond the cradle—Amidst microplastics and the ongoing peril during pregnancy and neonatal stages: a holistic review. J Hazard Mater. 2024;469:133963. 10.1016/j.jhazmat.2024.133963 38461669

[63] Jia H, Liu S, Wang W et al. Microplastic exposure induces preeclampsia-like symptoms via HIF-1α/TFRC-mediated ferroptosis in placental trophoblast cells. Toxicology. 2025;516:154197. 10.1016/j.tox.2025.154197 40414414

[64] Huang W, Hu W, Fang M et al. Impacts of prenatal environmental exposures on fetal-placental-maternal bile acid homeostasis and long-term health in offspring. Ecotoxicol Environ Saf. 2024;283:116929. 10.1016/j.ecoenv.2024.116929 39213751

[65] Nilsson EE, Winchester P, Proctor C et al. Epigenetic biomarker for preeclampsia-associated preterm birth and potential preventative medicine. Environ Epigenetics. 2024;10:dvae022. 10.1093/eep/dvae022 PMC11602036 39606093

[66] Vaka VR, McMaster KM, Cunningham MW et al. Role of mitochondrial dysfunction and reactive oxygen species in mediating hypertension in the reduced uterine perfusion pressure rat model of preeclampsia. Hypertension. 2018;72:703–11. 10.1161/HYPERTENSIONAHA.118.11290 30012871 PMC6394841

[67] Li S, Wang Z, Vieira LA et al. Improving preeclampsia risk prediction by modeling pregnancy trajectories from routinely collected electronic medical record data. npj Digit Med. 2022;5:68. https://www.nature.com/articles/s41746-022-00612-x 35668134 10.1038/s41746-022-00612-x PMC9170686

[68] Layton AT. Artificial intelligence and machine learning in preeclampsia. Arterioscler Thromb Vasc Biol. 2025;45:165–71. 10.1161/ATVBAHA.124.321673 39744839

[69] Torres-Torres J, Espino-y-Sosa S, Martinez-Portilla R et al. A narrative review on the pathophysiology of preeclampsia. Int J Mol Sci. 2024;25:7569. https://www.mdpi.com/1422-0067/25/14/7569 39062815 10.3390/ijms25147569 PMC11277207

[70] Bukowska P, Bralewska M, Pietrucha T et al. Nutraceuticals as modulators of molecular placental pathways: their potential to prevent and support the treatment of preeclampsia. Int J Mol Sci. 2024;25:12167. https://www.mdpi.com/1422-0067/25/22/12167 39596234 10.3390/ijms252212167 PMC11594370

[71] George EM, Bakrania BA, Granger JP et al. Animal models used for investigating pathophysiology of preeclampsia and identifying therapeutic targets. In: Taylor RN, Conrad KP, Davidge ST *et al*. (eds.), Chesley’s Hypertensive Disorders in Pregnancy. Amsterdam: Elsevier, 2022, 435–47. https://linkinghub.elsevier.com/retrieve/pii/B9780128184172000154

[72] Wu J, Ren C, Delfino RJ et al. Association between local traffic-generated air pollution and preeclampsia and preterm delivery in the South Coast Air Basin of California. Environ Health Perspect. 2009;117:1773–79. 10.1289/ehp.0800334 20049131 PMC2801174

